# Low-Temperature Vapor-Phase Synthesis of Single-Crystalline Gold Nanostructures: Toward Exceptional Electrocatalytic Activity for Methanol Oxidation Reaction

**DOI:** 10.3390/nano9040595

**Published:** 2019-04-10

**Authors:** Siyeong Yang, Kkotchorong Park, Bongsoo Kim, Taejoon Kang

**Affiliations:** 1 Department of Chemistry, KAIST, Daejeon 34141, Korea; yy1633@kaist.ac.kr (S.Y.); smile_cho@kaist.ac.kr (K.P.); 2 Bionanotechnology Research Center, KRIBB, Daejeon 34141, Korea; 3 Department of Nanobiotechnology, KRIBB School of Biotechnology, UST, Daejeon 34113, Korea

**Keywords:** low temperature, vapor-phase synthesis, Au nanostructures, electrocatalyst, methanol oxidation reaction

## Abstract

Au nanostructures (Au NSs) have been considered promising materials for applications in fuel cell catalysis, electrochemistry, and plasmonics. For the fabrication of high-performance Au NS-based electronic or electrochemical devices, Au NSs should have clean surfaces and be directly supported on a substrate without any mediating molecules. Herein, we report the vapor-phase synthesis of Au NSs on a fluorine-doped tin oxide (FTO) substrate at 120 °C and their application to the electrocatalytic methanol oxidation reaction (MOR). By employing AuCl as a precursor, the synthesis temperature for Au NSs was reduced to under 200 °C, enabling the direct synthesis of Au NSs on an FTO substrate in the vapor phase. Considering that previously reported vapor-phase synthesis of Au NSs requires a high temperature over 1000 °C, this proposed synthetic method is remarkably simple and practical. Moreover, we could selectively synthesize Au nanoparticles (NPs) and nanoplates by adjusting the location of the substrate, and the size of the Au NPs was controllable by changing the reaction temperature. The synthesized Au NSs are a single-crystalline material with clean surfaces that achieved a high methanol oxidation current density of 14.65 mA/cm^2^ when intimately supported by an FTO substrate. We anticipate that this novel synthetic method can widen the applicability of vapor-phase synthesized Au NSs for electronic and electrochemical devices.

## 1. Introduction

Au nanostructures (Au NSs) have been widely used for applications in electronics, electrochemistry, plasmonics, biomedical sensing, etc. [1,2,3,4,5]. In particular, Au NSs have been considered promising catalysts for the methanol oxidation reaction (MOR) because Au NSs do not form poisoning intermediates during electrocatalytic reactions [6,7,8,9]. Therefore, various kinds of Au NSs have been synthesized and applied to electrocatalytic reactions to improve catalytic activities [10,11,12,13]. According to previous studies, the intrinsic properties of Au NSs, such as their size, shape, and lattice plane, highly influence the catalytic activity of Au NSs [14,15,16,17]. In addition, the interface structure between Au NSs and the supporting electrode is critical to the improvement of the electrocatalytic activity since the interface structure significantly affects electron transfer from Au NSs to the conductive substrate and vice versa [18,19,20]. Moreover, clean Au NS surfaces are important to increase the number of effective collisions between reactants and catalytic active sites on the Au NS surface [21,22,23,24]. Thus, an ideal electrocatalytic Au NS should have a well-defined morphology, crystallinity, clean surfaces, and direct interface with a substrate.

For the synthesis of Au NSs, solution-phase synthetic methods have been commonly employed because these methods enable large-scale synthesis of Au NSs and control of the NS morphology by using ligand molecules [25,26,27]. However, ligand molecules often inhibit the catalytic activity of Au NSs since ligand molecules can interrupt electron transfer and collision with reactants [23,28,29,30,31]. In addition, the agglomeration of Au NSs can also lower the efficiency of catalytic reactions [32,33,34]. Recently, vapor-phase synthesis of Au NSs has emerged as a promising synthesis technique because vapor-phase synthetic methods can produce surfactant-free, single-crystalline, ultraclean, ultraflat, and morphology-tunable Au NSs [35,36,37,38,39]. Although the Au NSs synthesized in a vapor phase exhibit excellent physicochemical properties, they have rarely been applied to electrocatalytic reactions because vapor-phase-grown Au NSs are mainly deposited on nonconductive and temperature-stable substrates. The vapor-phase growth temperature of Au NSs is high (over 1000 °C) and, thus, versatile electrically conductive substrates, such as indium tin oxide and fluorine doped tin oxide (FTO), cannot be utilized [35,40]. Some papers reported the lowed temperature (below 1000 °C) [41,42]. While the adoption of an organometallic compound as a precursor allows a reaction temperature below ~500 °C, the decomposition of the organometallic compound generates byproducts that adsorb on the Au surfaces [43]. If we can routinely synthesize high-quality Au NSs on a conductive substrate in a vapor phase, the product may be a promising Au NS-based catalytic electrode.

Herein, we report a novel vapor-phase synthesis of Au NSs in the temperature range from 120 to 200 °C and the electrocatalytic MOR application of Au NSs. The use of AuCl as a precursor enabled an organic-molecule-free synthesis of Au NSs on an FTO substrate at low temperature. The synthetic reactions were investigated by X-ray photoelectron spectroscopy (XPS) analysis, which confirmed the disproportionation reaction of AuCl. We found that Au nanoparticles (NPs) and nanoplates could be selectively synthesized depending on the location of the substrate, and the size of the Au NPs could be controlled by the reaction temperature. Importantly, Au NPs on an FTO substrate were employed as a catalytic electrode for the MOR, and the electrode exhibited a current density of 14.65 mA/cm^2^, which is 33 times higher than that obtained with commercial Au NPs on an FTO substrate. This proposed vapor-phase synthetic method for Au NSs enables the direct growth of single-crystalline and clean Au NSs on a desired substrate and is a promising approach for the development of electrocatalysts and electrochemical sensing platforms based on Au NSs.

## 2. Materials and Methods

### 2.1. Synthesis of Au NPs on an FTO Substrate

The FTO coated glass slides, an electrically conductive substrate, were purchased from Sigma-Aldrich (No. 735159, Saint Louis, MO, USA). The FTO substrates were sonicated in acetone, ethanol, and water for 30 min, washed with acetone, and dried by N_2_. To synthesize Au NPs on a clean FTO substrate, 1.5 mg of fresh AuCl powder (99.9%, Aldrich) was placed in the center of the heating zone in a furnace with a 1 inch diameter inner quartz tube, and an FTO substrate was placed 15–20 mm from the AuCl powder toward the downstream side. Next, the furnace was heated to the desired temperature and maintained for 30 min under a flow of Ar (150 sccm). The pressure of the chamber was 0.7 Torr.

### 2.2. Synthesis of Au Nanoplates on an FTO Substrate

The FTO substrates were sonicated in acetone, ethanol, and water for 30 min, washed with acetone, and dried by N_2_. To synthesize Au nanoplates on a clean FTO substrate, 1.5 mg of fresh AuCl powder was placed in the center of the heating zone in a furnace, and an FTO substrate was placed 3–5 mm from the AuCl powder toward the downstream side. Next, the furnace was heated to 200 °C and maintained at that temperature for 30 min under a flow of Ar (150 sccm). The pressure of the chamber was 0.7 Torr.

### 2.3. Cyclic Voltammetry (CV) Measurements

All electrochemical analyses were carried out in the ambient atmosphere using a three-electrode cell consisting of vapor-phase-grown Au NP/FTO or colloidal Au NP/FTO as the working electrode, Pt wire as the auxiliary electrode, and a saturated calomel electrode (SCE) as the reference electrode. For vapor-phase-grown Au NP/FTO, the FTO substrate was partially coated with an epoxy resin after the reaction in furnace system to prevent contact between the solution and the electrode clamp. The vapor-phase-grown Au NP/FTO electrode was prepared as described above. The colloidal Au NP/FTO electrode was prepared by dropwise evaporation of Au NPs stabilized suspension in citrate buffer (20 nm, Aldrich) on an FTO substrate, rinsing with water, and drying with N_2_. The electrochemical active surface area of the Au NPs was measured in 3 mL of a 0.1 M KOH (Junsei, Tokyo, Japan) aqueous solution by cyclic scanning from −0.1 to 0.5 V (for vapor-phase Au NP/FTO) or 0.45 V (for colloidal Au NP/FTO) (vs. SCE, Rosemead, CA, USA). Before measurements, the 0.1 M KOH and methanol solutions were purged with N_2_ for 30 min. The electrooxidation of methanol was measured by cyclic scanning from −0.15 to 0.5 V (vs. SCE).

### 2.4. Instrumentation

Field-emission scanning electron microscopy (SEM) images were obtained using a Nova 230 (CA, USA). Transmission electron microscopy (TEM) images, high-resolution TEM (HR-TEM) images, and electron diffraction patterns were taken on a TECNAI TF30 ST transmission electron microscope (Hilsboro, OR, USA) operated at 300 kV. X-ray diffraction (XRD) spectra were obtained with a D/MAX-2500 instrument (RIGAKU, Auburn Hills, MI, USA). The cross-sectional TEM image of the Au nanoplate was obtained using a Helios Nanolab 450 F1 microscope (FEI company, Hillsboro, OR, USA). X-ray photoelectron spectroscopy (XPS) spectra were obtained from K-alpha (Thermo VG Scientific, Waltham, MA, USA). Cyclic voltammetry (CV) was recorded using a computer-controlled CHI643B electrochemical analyzer (Austin, TX, USA).

## 3. Results and Discussion

**Figure 1:**
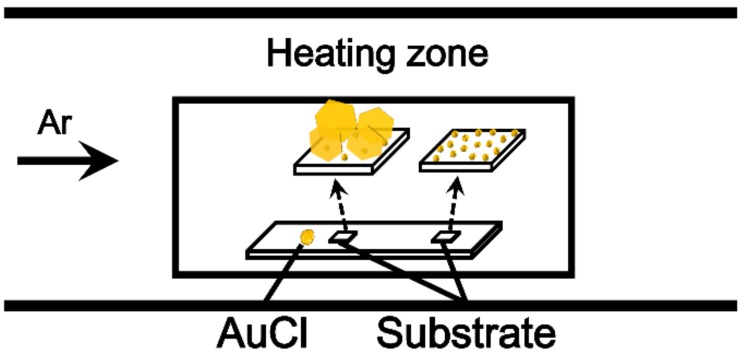
Schematic illustration of the vapor-phase synthesis of Au nanostructures (NSs) at low temperature. When the substrate was placed 15–20 mm away from AuCl, Au NPs were grown on the substrate. When the substrate was placed 3–5 mm away from AuCl, Au nanoplates were obtained on the substrate.

Figure 1 is a schematic illustration of the vapor-phase synthesis of Au NSs. AuCl powder was placed in the heating zone of a horizontal quartz tube furnace system, and the FTO substrate was positioned 3–20 mm away from the AuCl powder. For the synthesis of Au NSs, the system was heated to 120–200 °C under an Ar gas flow of 150 sccm. The pressure of the chamber was maintained at 0.7 Torr. After reacting for 30 min, single-crystalline Au NSs were obtained on an FTO substrate. As shown in Figure 1, we could synthesize single-crystalline Au nanoplates and Au NPs. The morphology can be controlled by adjusting the distance between the AuCl precursor and the FTO substrate. When the substrate was placed 15–20 mm away from the AuCl, only Au NPs were grown on the substrate. When the substrate was closer (3–5 mm) to the precursor, Au nanoplates were obtained on the substrate. The previous literature suggested that the flux of the precursor can affect the morphology of noble metal NSs during vapor-phase growth [35]. In this experiment, the morphology of the Au NSs can also be controlled by the flux of the AuCl.

Au commonly exists in nature as Au^0^, Au^1+^, and Au^3+^ [44]. Previously, our group evaporated a Au slug (Au^0^) at temperatures greater than 1000 °C and successfully synthesized several kinds of Au NSs [40]. Wang et al. employed HAuCl_4_, in which Au is in a +3 oxidation state, as a starting material for the vapor-phase synthesis of Au NSs [45]. Although the use of HAuCl_4_ lowered the reaction temperature to ~500 °C, the temperature was still too high for the synthesis of Au NSs on an FTO substrate. To synthesize Au NSs on an FTO substrate in a vapor phase, we used AuCl as a precursor because the disproportionation reaction of AuCl can occur under mild conditions [46,47,48]. As a result, single-crystalline Au NPs and nanoplates were successfully synthesized at temperatures below 200 °C.

**Figure 2:**
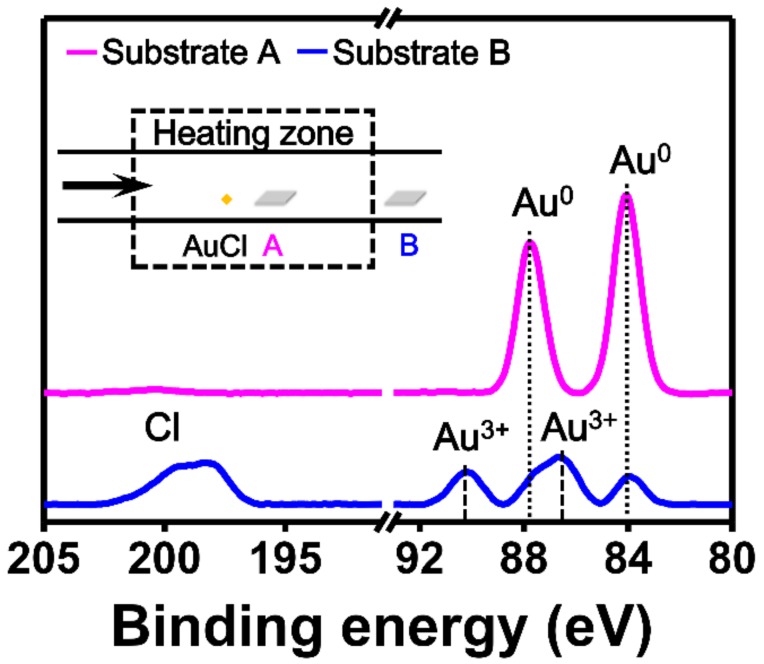
X-ray photoelectron spectroscopy (XPS) spectra obtained for two substrates placed in or out of the heating zone. Substrate A was positioned approximately 10 mm away from AuCl, and substrate B was positioned approximately 250 mm away from the precursor (inset). The binding energy peaks corresponding to Au^0^ were measured on substrate A (magenta). Binding energy peaks corresponding to Au^3+^ and Cl were measured on substrate B (blue).

To investigate whether AuCl disproportionated to produce metallic Au (Au^0^) during the experiment, we analyzed the XPS spectra of the substrates after chemically reacting in the experimental system. Figure 2 shows the XPS spectra obtained for the substrates placed in or out of the heating zone. Substrate A was positioned approximately 10 mm from the AuCl powder, and substrate B was positioned approximately 250 mm from the precursor (inset of Figure 2). For substrate A, only the binding energy peaks corresponding to metallic Au (Au^0^) were measured. For substrate B, peaks corresponding to Au^3+^ and Cl were also observed [49,50]. The XPS results suggest that AuCl can supply Au atoms to substrate A by the disproportionation reaction of AuCl (3AuCl → 2Au + AuCl_3_) [38,51]. The resultant 2 Au atoms (Au^0^) were deposited on the FTO substrate immediately after the disproportionation reaction occurred because the reaction temperature of this synthetic method was below 200 °C. The remaining AuCl_3_ vapor was carried by the carrier gas flow and condensed on substrate B. This growth mechanism indicates that the amount of Au^0^ can be varied by changing the substrate location and the reaction temperature. Therefore, we could selectively synthesize Au NPs and nanoplates by adjusting the substrate location, and we could control the size of the NPs by changing the reaction temperature.

**Figure 3:**
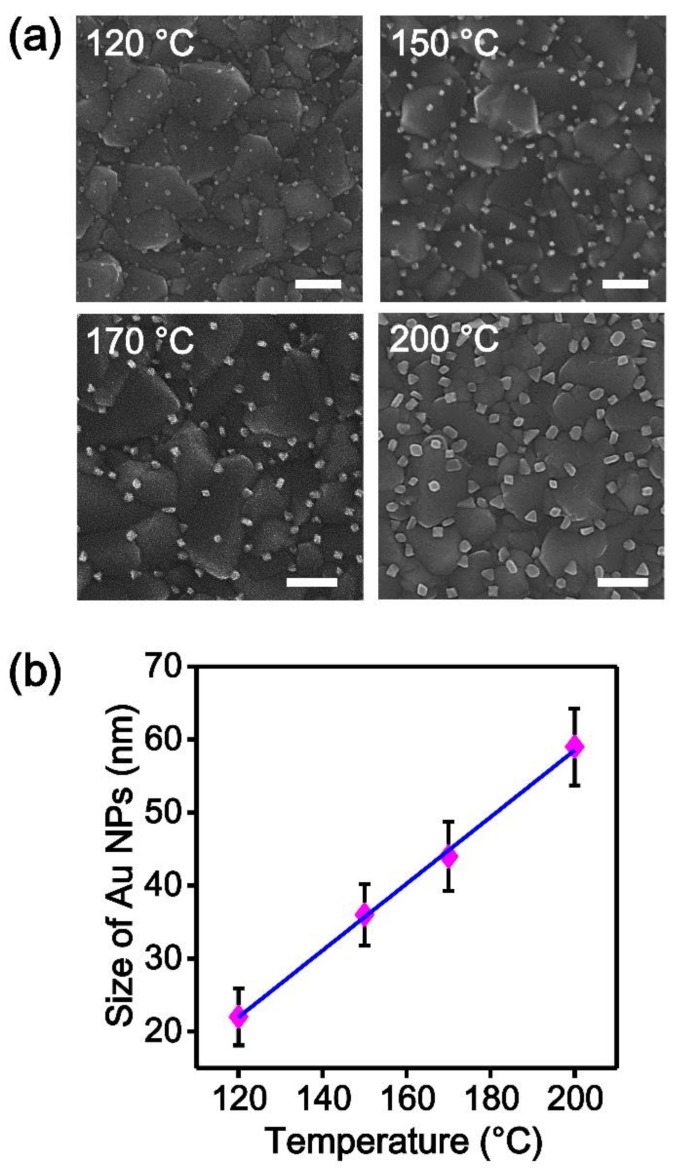
(**a**) Scanning electron microscopy (SEM) images of the Au nanoparticles (NPs) grown on fluorine-doped tin oxide (FTO) substrates. The size of the Au NPs increased as the reaction temperature increased from 120 to 200 °C. Scale bar is 200 nm. (**b**) Plot of the average size of the Au NPs versus the reaction temperature. The average size of the Au NPs increased from 23 ± 3.9 nm to 36 ± 4.2 nm, 44 ± 4.7 nm, and 59 ± 5.3 nm with the increase in the reaction temperature. The linear fit line is also shown (blue). Data represent the mean plus standard deviation from twenty measurements.

Figure 3a shows SEM images of the Au NPs grown on FTO substrates. The as-synthesized Au NPs were well dispersed on the substrates without aggregation. The X-ray diffractogram also suggests that crystalline Au NSs were synthesized on an FTO substrate (Figure S1) [52]. Interestingly, the SEM images show that the size of the Au NPs increased as the reaction temperature increased. Figure 3b is the plot of the average size of the Au NPs versus the reaction temperature. The average size of the Au NPs linearly increased from 23 ± 3.9 nm to 36 ± 4.2 nm, 44 ± 4.7 nm, and 59 ± 5.3 nm as the reaction temperature increased from 120 to 150, 170, and 200 °C, respectively. The linear fit line was determined to be *y* = 0.457*x* − 32.90 with an *R*^2^ value of 0.998. As the reaction temperature increases, more of the AuCl precursor may be vaporized, and thus, a more concentrated AuCl vapor could be supplied to the substrate, leading to an increase in the Au NP size.

**Figure 4:**
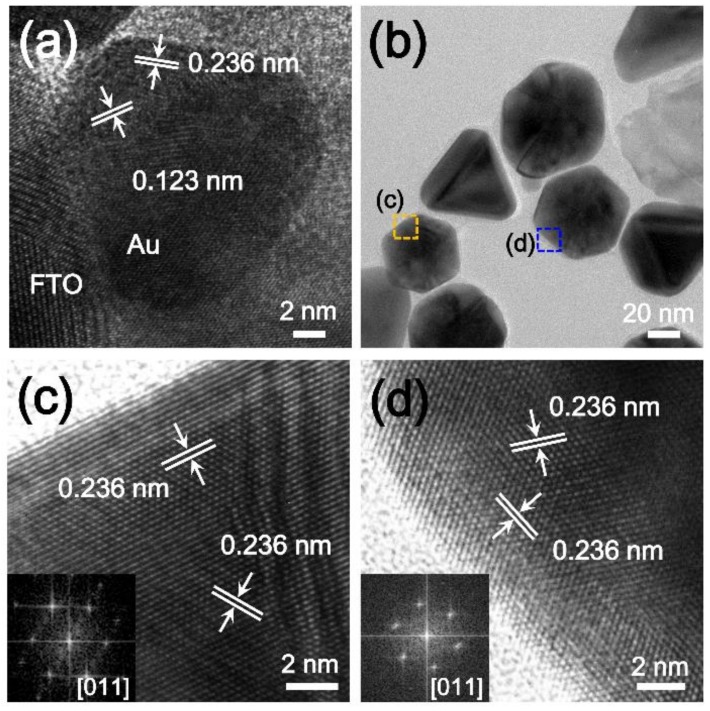
(**a**) Cross-sectional high-resolution transmission electron microscopy (HR-TEM) image of Au NPs on an FTO substrate. The Au NPs directly interfaced with the FTO substrate without any space; (**b**) TEM image of Au NPs detached from the FTO substrate; (**c**,**d**) HR-TEM images of Au NPs obtained from the (**c**) orange and (**d**) blue boxes of (**b**). Insets are fast Fourier transformation (FFT) patterns of the Au NPs.

The Au NPs synthesized on an FTO substrate were further investigated by a TEM analysis. First, we obtained a cross-sectional HR-TEM image of a Au NP on an FTO substrate using focused ion beam milling (Figure 4a). The cross-sectional HR-TEM image clearly showed that the Au NP directly interfaced with the FTO substrate without any space. This perfect contact can ensure excellent electron- transfer between the Au NPs and the substrate [18,19]. We also obtained a TEM image of Au NPs that were detached from the substrate after sonication for 30 min (Figure 4b–d). The HR-TEM analysis results suggested that the planes of the polyhedral Au NPs were uniformly enclosed by the Au (111) lattice plane. Moreover, the fast Fourier transformation (FFT) patterns clearly showed the single-crystalline nature of the Au NPs (insets of Figure 4c,d).

**Figure 5:**
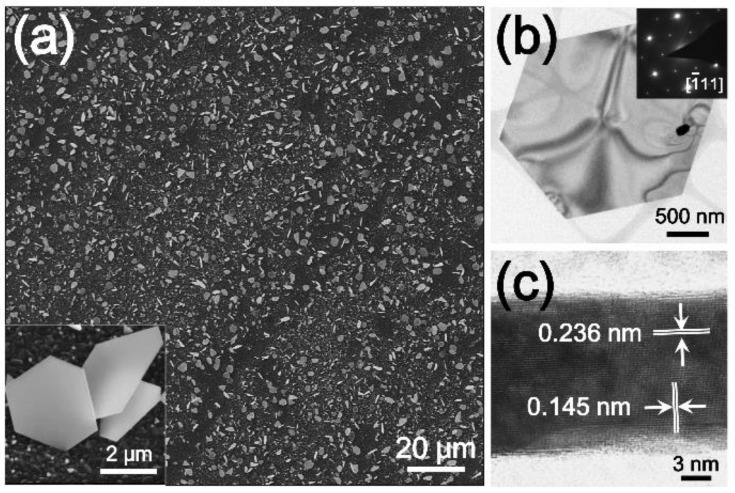
(**a**) SEM image of free-standing Au nanoplates synthesized on an FTO substrate. The inset is a magnified SEM image of the Au nanoplates; (**b**) TEM image and selected-area electron diffraction (SAED) pattern (inset) of the Au nanoparticles; and (**c**) cross-sectional HR-TEM image of the Au nanoplate.

Figure 5a shows the SEM image of free-standing Au nanoplates synthesized on an FTO substrate. The Au nanoplates were densely grown on the substrate and have well-defined shapes. The TEM image and selected-area electron diffraction (SAED) pattern of the nanoplate indicated that there are diffraction pattern originated from 1/3{422} reflection (inner small points in Figure 5b) [53]. The spots are displayed due to the stacking faults parallel to the (111) basal plane of nanoplate. Additionally, the cross-sectional HR-TEM image of the Au nanoplate confirmed that the thickness of the Au nanoplates was measured to be only ~15 nm, which is much thinner than the thickness of previous Au nanoplates (average thickness of 100 nm) synthesized in a vapor phase at high temperature (Figure 5c). The surface planes of the Au nanoplates were arranged by a Au (111) crystal plane, which corresponded to the TEM results of the Au NPs. Because Au (111) is the most stable crystal plane among the Au crystal planes, the Au NSs were faceted with the Au (111) planes during the synthesis. Previously, it was reported that the disproportionation reaction of AuCl can be autocatalyzed on a Au surface [44]. Therefore, the growth of the Au nanoplates could be induced by the autocatalyzed reaction of AuCl under the conditions of a high AuCl vapor flux. During the Au nanoplate synthesis reaction, Au nuclei particles may first form on the substrate. Then, the AuCl vapor collides with the Au nuclei particles and catalyzes the disproportionation of AuCl. Since AuCl disproportionation is more active on the high-energy crystal plane of Au, an enlargement of the Au (111) plane might be favored, and Au nanoplates may be formed. The proposed synthetic method can provide single-crystalline Au NSs without surfactants and allows us to control the morphology and size of the NSs even at a low temperature of 200 °C; therefore, this method may open new routes for the vapor-phase synthesis of noble metal NSs and their applications.

**Figure 6:**
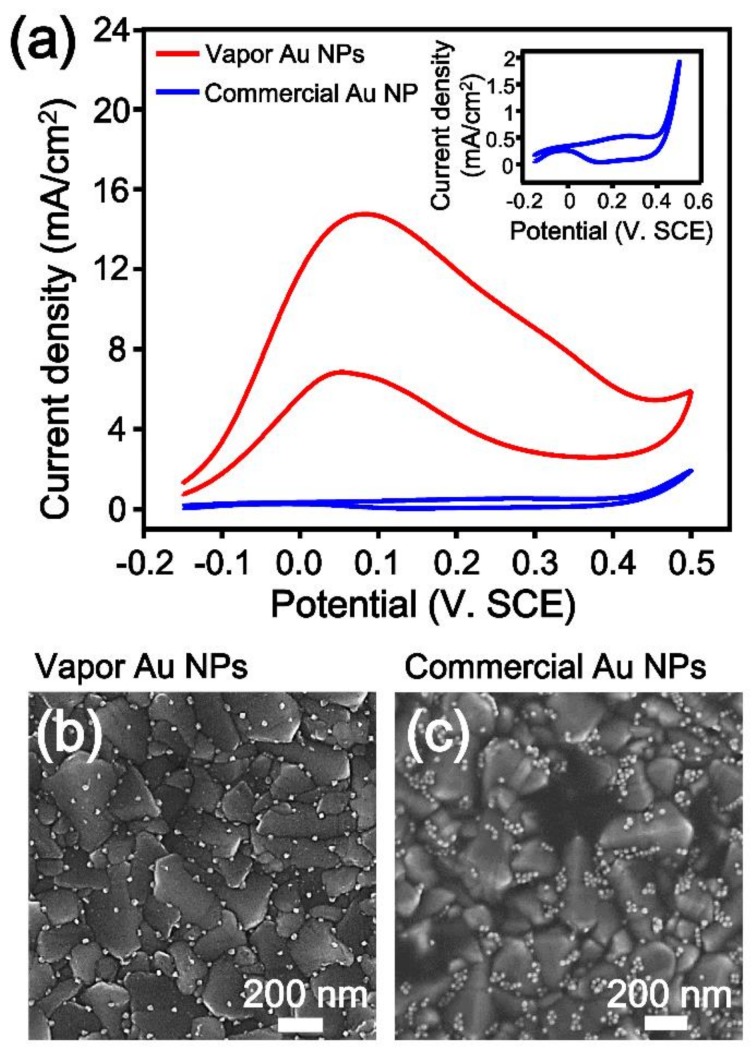
(**a**) Cyclic voltammetry (CV) curves obtained with vapor-phase-grown Au NP electrodes (red) and commercial Au NP electrodes (blue) in a solution of 0.1 M KOH + 1.5 M methanol (scan rate = 50 mV/s). Inset is the magnified CV curve for commercial Au NPs; (**b**,**c**) SEM images of (**b**) vapor-phase-grown Au NP electrodes and (**c**) commercial Au NP electrodes after the CV measurements. The vapor-phase-grown Au NPs were well dispersed on the FTO substrate, but the commercial Au NPs were slightly aggregated.

The electrocatalytic oxidation of methanol has been widely studied for the construction of direct methanol fuel cells [11,54,55]. During the past decade, Pt has been commonly employed as a catalytic material for the electrooxidation of methanol [56,57,58]. Although Pt-based catalysts show high catalytic activity for the MOR in acidic media, they often suffer from strong chemisorption of poisoning intermediates during the reaction [59,60]. Au-based catalysts exhibit high activity in alkaline media and are free from the formation of poisoning intermediates during the electrooxidation of methanol [9,44,61,62]. Therefore, many studies have reported using Au NSs as catalysts for the electrocatalytic oxidation of methanol [10,11,63,64,65]. Since the Au NPs prepared in this study have the properties necessary to function as an electrocatalyst for the MOR, such as clean surfaces, a direct interface with an FTO substrate, and single crystallinity, we examined the electrocatalytic activity of the Au NPs for the MOR. By employing vapor-phase-grown Au NPs on an FTO substrate as a catalytic electrode, CV curves for the MOR were measured in a 0.1 M KOH + 1.5 M methanol solution. For comparison, we also prepared a catalytic electrode by dropwise evaporation of commercial colloidal Au NPs on an FTO substrate. Since the size of the Au NPs can significantly influence the catalytic activity, two kinds of Au NPs were prepared with the same average diameter of 20 nm. Additionally, a bare FTO substrate was tested as an electrode to verify that the bare FTO substrate shows no electrocatalytic activity for the MOR (Figure S2). The red line in Figure 6a is the CV curve for the MOR with the vapor-phase-grown Au NPs, and the blue line is the CV curve for the commercial Au NPs. The inset is a magnified image of the CV curve of the commercial Au NPs. The oxidation current density and onset potential for the MOR clearly indicate the outstanding electrocatalytic activity of the vapor-phase-grown Au NPs. The oxidation current density of the Au NPs was calculated by dividing the measured currents by the electrochemical active surface areas (ECSAs) of the electrodes [66]. The ECSAs of the vapor-phase grown and commercial Au NPs were obtained from the CV curves in a 0.1 M KOH solution (Figure S3). It is noteworthy that the oxidation current density of the vapor-phase-grown Au NPs (14.65 mA/cm^2^) was 33 times higher than that of the commercial Au NPs (0.44 mA/cm^2^). Furthermore, the vapor-phase-grown Au NPs showed an approximately 0.15 V lower onset potential than the commercial Au NPs, meaning that the oxidation of methanol started earlier when the vapor-phase-grown Au NPs were used.

After the CV measurements, SEM images of catalytic electrodes were obtained (Figure 6b,c). The vapor-phase-grown Au NPs were well dispersed on an FTO substrate, but the commercial Au NPs were slightly aggregated. The agglomeration would contribute to the low electrocatalytic activity of the commercial Au NPs [67,68]. To the best of our knowledge, the oxidation current density of 14.65 mA/cm^2^ obtained in this experiment is the highest value among those measured in the same media using Au-based electrodes, including self-supported nanoporous Au film electrodes [11], polycrystalline Au electrodes [13], and nanoporous Au electrodes prepared by dealloying Au-Ag [10]. The excellent catalytic activity of the Au NPs can be attributed to the combination of their clean surfaces [21,728, facets well-arranged by the Au (111) plane [69,70], and intimate contact with the FTO substrate. Consequently, we expect that this Au NS-based electrode can be a versatile tool for various catalytic and electrochemical applications.

## 4. Conclusions

In conclusion, we report the low-temperature vapor-phase synthesis of single-crystalline Au NSs on a conductive substrate and their electrocatalytic application to the MOR. The use of AuCl as a precursor enabled the synthesis of Au NSs at temperatures below 200 °C through the disproportionation reaction of AuCl. In this method, Au nanoplates and Au NPs can be selectively synthesized by adjusting the location of the substrate. Moreover, the size of the Au NPs is controllable by varying the reaction temperature. The SEM, TEM, and XPS analyses elucidated the growth mechanism of the Au NSs in the low-temperature vapor phase. Surprisingly, the vapor-phase-grown Au NPs had an oxidation current density of 14.65 mA/cm^2^ for the electrocatalytic MOR. This value is 33 times higher than that obtained from commercial Au NPs. Additionally, the vapor-phase-grown Au NPs showed a 0.15 V lower onset potential than the commercial Au NPs, which indicates the exceptional catalytic performance of the vapor-phase-grown Au NP electrode. We anticipate that this new synthetic approach could expand vapor-phase synthesis of noble metal NSs and their applications to a variety of electrocatalytic or electrochemical reactions.

## Supplementary Materials

The following are available online at https://www.mdpi.com/2079-4991/9/4/595/s1; Figure S1: XRD spectrum of the Au NPs synthesized on an FTO substrate, Figure S2: CV curves obtained with a bare FTO substrate in the absence (blue) and presence (red) of 1.5 M methanol in 0.1 M KOH (scan rate = 50 mV/s), Figure S3: CV curves obtained with vapor-phase-grown Au NP electrodes (red) and commercial Au NP electrode (blue) in a solution of 0.1 M KOH (scan rate = 50 mV/s).
