# Supplementary material for: Low-Temperature Vapor-Phase Synthesis of Single-Crystalline Gold Nanostructures: Toward Exceptional Electrocatalytic Activity for Methanol Oxidation Reaction
Source: Nanomaterials (Basel). 2019 Apr 10;9(4):595. doi: 10.3390/nano9040595 (PMC6523424; doi:10.3390/nano9040595)
Supplement: Supplementary file 1 [file nanomaterials-09-00595-s001.pdf]

Supporting Information

Low-Temperature Vapor-Phase Synthesis of Single-Crystalline Gold Nanostructures: Toward Exceptional Electrocatalytic Activity for Methanol Oxidation Reaction

Siyeong Yang [1], Kkotchorong Park [1], Bongsoo Kim [1,*] and Taejoon Kang [2,3,*]

[1] Department of Chemistry, KAIST, Daejeon 34141, Korea; yy1633@kaist.ac.kr (S.Y.); smile_cho@kaist.ac.kr (K.P.)

[2] Bionanotechnology Research Center, KRIBB, Daejeon 34141, Korea

[3] Department of Nanobiotechnology, KRIBB School of Biotechnology, UST, Daejeon 34113, Korea

* Correspondence: kangtaejoon@kribb.re.kr (T.K.); bongsoo@kaist.ac.kr (B.K.)

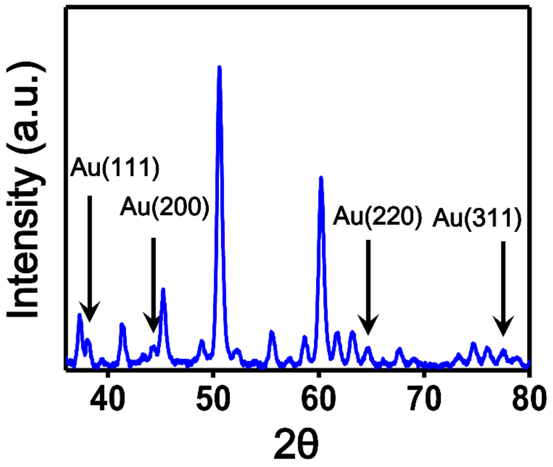

Figure S1. XRD spectrum of the Au NPs synthesized on an FTO substrate. The Au peaks are marked by arrows and the other peaks are signals related to the intrinsic substrate.

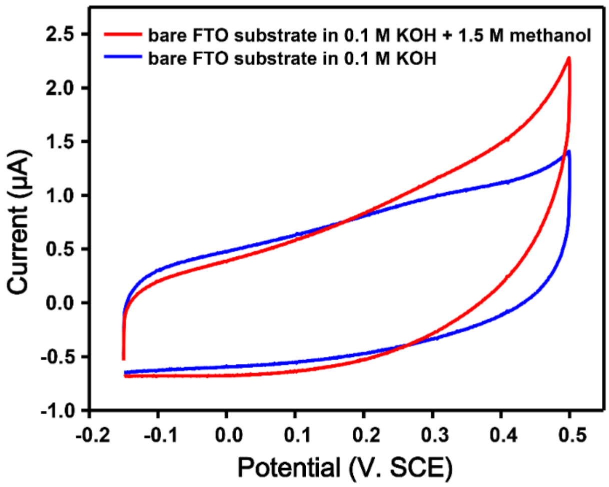

Figure S2. CV curves obtained with a bare FTO substrate in the absence (blue) and presence (red) of 1.5 M methanol in 0.1 M KOH (scan rate = 50 mV/s).

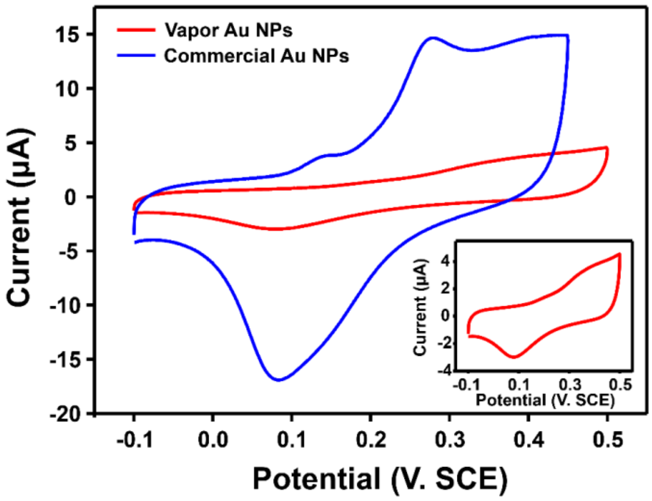

Figure S3. CV curves obtained with vapor-phase-grown Au NP electrodes (red) and commercial Au NP electrodes (blue) in a solution of 0.1 M KOH (scan rate = 50 mV/s). Inset is the magnified CV curve for the vapor-phase Au NPs. The electrochemical active surface area was calculated by integrating the consumed charge in the reduction curves.
